# Supplementary material for: RHOJ controls EMT-associated resistance to chemotherapy
Source: Nature. 2023 Mar 22;616(7955):168–75. doi: 10.1038/s41586-023-05838-7 (PMC10076223; doi:10.1038/s41586-023-05838-7)
Supplement: Supplementary file 1 — Supplementary Figs. 1 and 2. Supplementary Fig. 1: unprocessed original scans of Western blot analysis. Each panel indicates the figure to which the full membranes belong. Molecular mass size standards are indicated on each membrane. Supplementary Fig. 2: unprocessed original scans of western blot analyses. Each panel indicates the figure to which the full membranes belong (a–e). Molecular mass size standards are indicated on each membrane. Unprocessed original gel picture of PCR genotype analysis (f). [file 41586_2023_5838_MOESM1_ESM.pdf]

nature portfolio

https://doi.org/10.1038/s41586-023-05838-7

Supplementary information

RHOJ controls EMT-associated resistance to chemotherapy

In the format provided by the authors and unedited

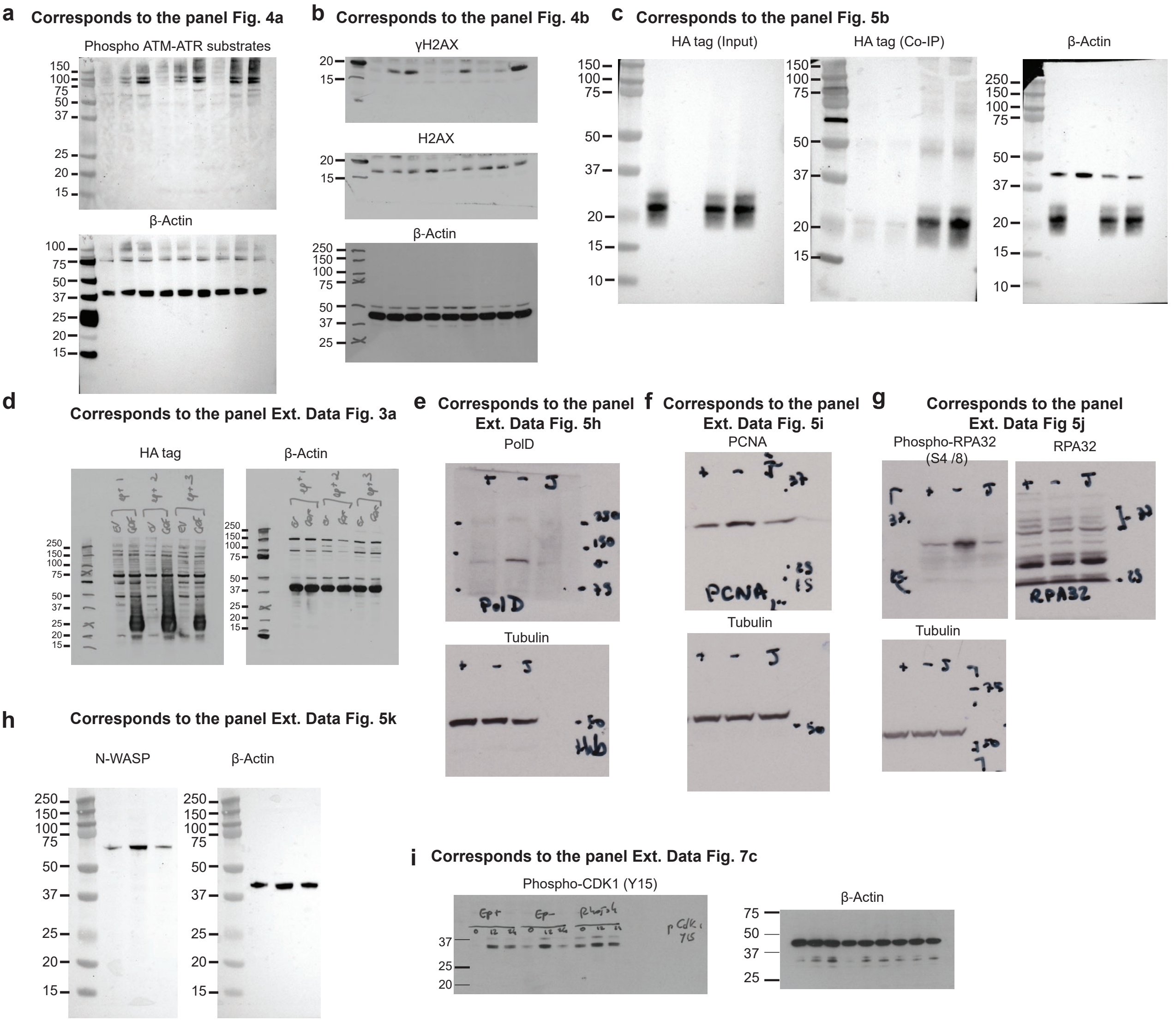

Supplementary Information Figure 1

a **Corresponds to the panel Ext. Data Fig. 7d**

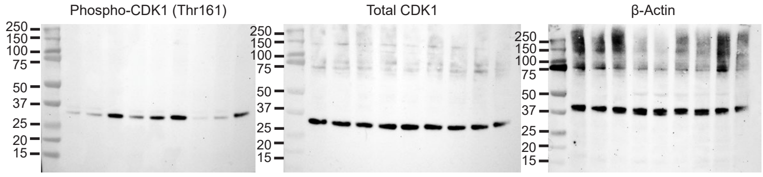

b **Corresponds to the panel Ext. Data Fig. 7e**

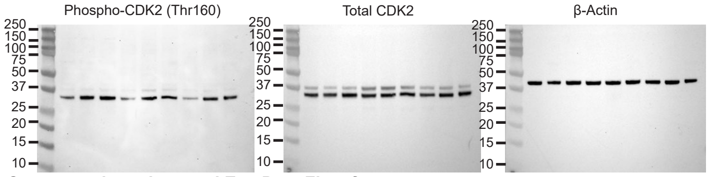

c **Corresponds to the panel Ext. Data Fig. 7f**

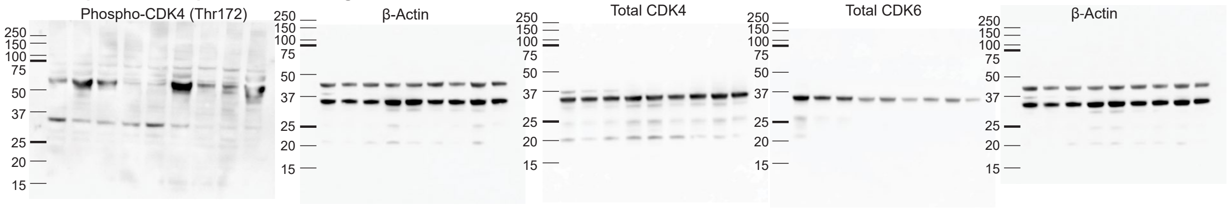

d **Corresponds to the panel Ext. Data Fig. 8c**

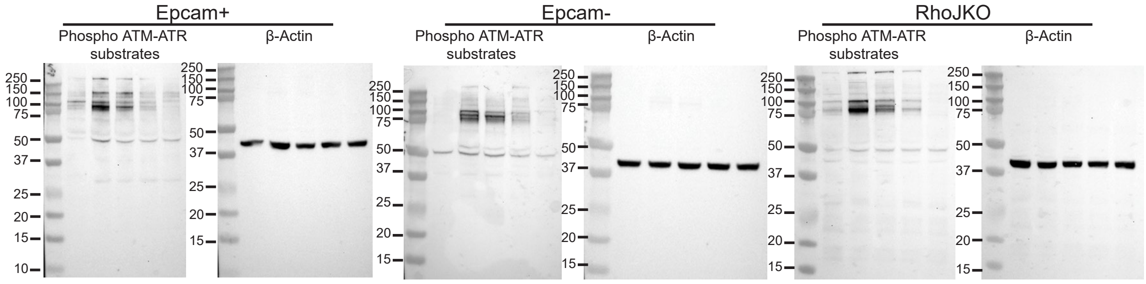

e **Corresponds to the panel Ext. Data Fig. 8h**

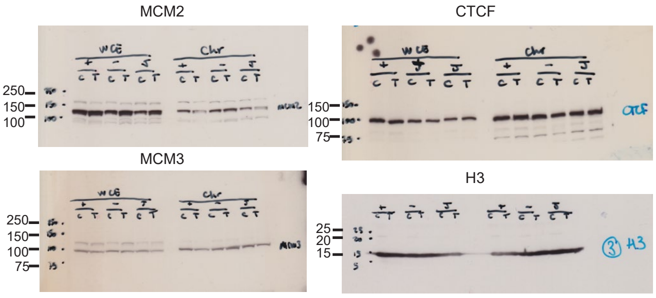

f **Corresponds to the panel Ext. Data Fig. 3e**

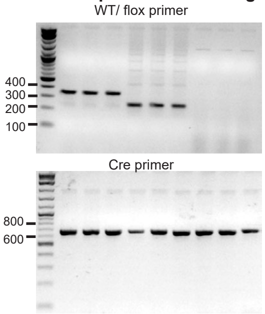

Supplementary Information Figure 2
