# Supplementary material for: RHOJ controls EMT-associated resistance to chemotherapy
Source: Nature. 2023 Mar 22;616(7955):168–75. doi: 10.1038/s41586-023-05838-7 (PMC10076223; doi:10.1038/s41586-023-05838-7)
Supplement: Supplementary file 2 — Reporting Summary [file 41586_2023_5838_MOESM2_ESM.pdf]

nature portfolio

Corresponding author(s): Cédric Blanpain

Last updated by author(s): Jan 31, 2023

Reporting Summary

Nature Portfolio wishes to improve the reproducibility of the work that we publish. This form provides structure for consistency and transparency in reporting. For further information on Nature Portfolio policies, see our Editorial Policies and the Editorial Policy Checklist.

Statistics

For all statistical analyses, confirm that the following items are present in the figure legend, table legend, main text, or Methods section.

n/a	Confirmed	
☐	☒	The exact sample size (*n*) for each experimental group/condition, given as a discrete number and unit of measurement
☐	☒	A statement on whether measurements were taken from distinct samples or whether the same sample was measured repeatedly
☐	☒	The statistical test(s) used AND whether they are one- or two-sided<br>*Only common tests should be described solely by name; describe more complex techniques in the Methods section.*
☒	☐	A description of all covariates tested
☐	☒	A description of any assumptions or corrections, such as tests of normality and adjustment for multiple comparisons
☐	☒	A full description of the statistical parameters including central tendency (e.g. means) or other basic estimates (e.g. regression coefficient) AND variation (e.g. standard deviation) or associated estimates of uncertainty (e.g. confidence intervals)
☐	☒	For null hypothesis testing, the test statistic (e.g. *F*, *t*, *r*) with confidence intervals, effect sizes, degrees of freedom and *P* value noted<br>*Give P values as exact values whenever suitable.*
☒	☐	For Bayesian analysis, information on the choice of priors and Markov chain Monte Carlo settings
☒	☐	For hierarchical and complex designs, identification of the appropriate level for tests and full reporting of outcomes
☒	☐	Estimates of effect sizes (e.g. Cohen's *d*, Pearson's *r*), indicating how they were calculated

Our web collection on statistics for biologists contains articles on many of the points above.

Software and code

Policy information about availability of computer code

Data collection	FACS ARIA III<br>Axiovision release 4.6<br>Zen Blue 3.3 (ZEISS)
Data analysis	STAR software (2.4.2a)<br>Prism 5 (Graphpad)<br>FACSDiva Software 8.0.1<br>FlowJo v10<br>MaxQuant (version 1.5.8.3) and (version 2.0.3.0)<br>Perseus Software (version 1.5.5.3)<br>Adobe Photoshop and Illustrator CS6 (Figure preparation)<br>R software (3.2.3)<br>ImageJ 1.53j<br>IBM-SPSS V28.0<br>Medcalc V 20<br>Huygens Professional 22.04 deconvolution software<br>CellProfiler 3.1.9 software

For manuscripts utilizing custom algorithms or software that are central to the research but not yet described in published literature, software must be made available to editors and reviewers. We strongly encourage code deposition in a community repository (e.g. GitHub). See the Nature Portfolio guidelines for submitting code & software for further information.

Data

Policy information about availability of data

All manuscripts must include a data availability statement. This statement should provide the following information, where applicable:

- Accession codes, unique identifiers, or web links for publicly available datasets
- A description of any restrictions on data availability
- For clinical datasets or third party data, please ensure that the statement adheres to our policy

The mass spectrometry proteomics data have been deposited to the ProteomeXchange Consortium via the PRIDE83 partner repository with the dataset identifier PXD025737 and the dataset identifier PXD038278.
The accession number for the RNA sequencing reported in this paper is GEO: GSE205985.

Field-specific reporting

Please select the one below that is the best fit for your research. If you are not sure, read the appropriate sections before making your selection.

☒ Life sciences ☐ Behavioural & social sciences ☐ Ecological, evolutionary & environmental sciences

For a reference copy of the document with all sections, see nature.com/documents/nr-reporting-summary-flat.pdf

Life sciences study design

All studies must disclose on these points even when the disclosure is negative.

Sample size	Samples sizes are indicated in the figures for each experiment. Samples sizes for experiments involving mice was determined accordingly to the protocols 434N and 663N, stating the number of mice used for experiments , should be reduced to the minimum as soon as the result is reproducible within each experiment. For in vitro experiments, samples sizes were determined based on previous experience in the lab and previously published studies of a similar nature (Latil Cell Stem Cell 2017, Pastushenko Nature 2020, Ibarra PNAS 2008, Lamm Nature Cell Biology 2020, Murai Molecular Cell 2018, Schrank Nature 2018) . No statistical methods were used to predetermine sample size.
Data exclusions	No data were excluded from the analysis
Replication	Each experiment was repeated at least three times to confirm reproducibility of findings, except for RNAseq analysis for which experiment was repeated twice. n is described in figure legends.
Randomization	For experiments involving cell culture, all tumor cell types (Epcam+, Epcam- and RhoJ KO Epcam- tumor cells and human MDA-MB231) were treated with all the drugs so no allocation in groups or randomization was required. For in vivo studies on primary mouse models , the animals were selected according to their correct genotypes. The mice were induced with Tamoxifen injection 28-35 days after birth and the mice developed tumors in 2-3 months thus minimizing the difference in age of different animals used. When tumor size reached between 2 and 5 mm3, mice were treated with chemotherapy injected intraperitoneally and were compared with tumors developed in mice of the same genotype injected with physiological serum.
Blinding	Investigators were blinded to mouse and cell line genotypes or treatment conditions during experiments, for performing sample analysis, imaging and quantification.

Reporting for specific materials, systems and methods

We require information from authors about some types of materials, experimental systems and methods used in many studies. Here, indicate whether each material, system or method listed is relevant to your study. If you are not sure if a list item applies to your research, read the appropriate section before selecting a response.

Materials & experimental systems

n/a	Involved in the study	
☐	☒	Antibodies
☐	☒	Eukaryotic cell lines
☒	☐	Palaeontology and archaeology
☐	☒	Animals and other organisms
☒	☐	Human research participants
☒	☐	Clinical data
☒	☐	Dual use research of concern

Methods

n/a	Involved in the study	
☒	☐	ChIP-seq
☐	☒	Flow cytometry
☒	☐	MRI-based neuroimaging

Antibodies

Antibodies used

For immunostaining, the following primary antibodies were used: goat GFP (Abcam, ab6673, 1:500), chicken K14 (Thermo Fisher Scientific, #MA5-11599, 1:2000), rabbit Vimentin (Abcam, ab92547, 1:500), rabbit active caspase-3 (R&D, AF835, 1:600), rabbit 53bp1 (Novus, NB100-304, 1:200), mouse phospho-histone H2A.X (Ser139) (Millipore, 05-636, 1:500), mouse RAD51 (Santa-Cruz Biotechnology, sc-398587, 1:50), rat RPA32/RPA2 (Cell Signaling, #2208, 1:500), Rhodamine Phalloidin (Thermo Fisher Scientific, R415, 1:400), rabbit HA (Abcam, ab9110, 1:1000), mouse PCNA (Abcam, ab29, 1:1000), rat phospho-histone H3 (S28) (Abcam, ab10543, 1:2000), rabbit Ki67 (Abcam, ab15580, 1:400). Validated for IF by manufacturer.
For western blotting, the following primary antibodies were used: rabbit phospho-histone H2A.X (Ser139) (Cell Signaling, #2577, 1:800), rabbit histone H2A.X (Cell Signaling, #2595, 1:1000), rabbit phospho-ATM/ATR substrate (Cell Signaling, #9607, 1:750), rabbit beta-Actin (Abcam, ab8227, 1:2000), mouse HA (Roche, #11583816001, 1:1000), rabbit phospho -CDC2 (Tyr15) (Cell Signaling, #4539, 1:1000),), rabbit phospho-CDC2 (Thr161) (Cell Signaling, #9114, 1:1000), rabbit CDC2 (Cell Signaling, #77055, 1:1000), rabbit phospho-CDK2 (Thr160) (Cell Signaling, #2561, 1:1000), rabbit CDK2 (Cell Signaling, #18048, 1:1000), rabbit CDK4 (Abcam, ab199728, 1:1000), rabbit CDK6 (Cell Signaling, #3136, 1:1000), rabbit N-WASP (Cell Signaling, #4848, 1:1000), mouse POLD (Santa Cruz #sc-373731), mouse PCNA (Santa Cruz #sc-56), rat RPA32 (Cell Signalling #2208), rabbit phospho-RPA32 S4/S8 (Bethyl #A300-245A), rabbit CTCF (Millipore #07-729), mouse alpha-tubulin (Sigma #T9026), rabbit H3 (Abcam #ab1791). The following secondary antibodies were used: ECL anti-Rabbit, anti-rat or anti-mouse IgG conjugated with horseradish peroxidase (#NA934, #NA935, #NA931,GE Healthcare, 1:2000 or 1:5000). Validated for WB by manufacturer. Affinity-purified mouse monoclonal antibodies NB8-AD9 (WB/IP) raised against human phospho-CDK4 (Thr172) have been validated for WB by Coulonval et al. 2022(1:500). Rabbit MCM2 and rabbit MCM3 have been validated for WB and described by Mendez and Stillman, 2000.
For immunodetection of labeled tracks, the primary antibodies used are :for CldU, rat anti-BrdU # ab6326 Abcam; for IdU, mouse anti-BrdU #347580 BD Bioscience, the corresponding secondary antibodies used are anti-rat IgG AF594, #A-11007; anti-mouse IgG1 AF488, #A-21121; all from Molecular Probes. Mouse anti-ssDNA antibody was used to assess fiber integrity (#MAB3034 Millipore, secondary antibody anti-mouse IgG2a AF647, #A-21241 Molecular Probes).
Immunostaining for FACS analysis was performed using PE-conjugated anti-CD45 (clone 30F11,#103114, 1:100, eBioscience), PE-conjugated anti CD31 (clone MEC13.3; #102508, 1:100, BD PharMingen), and APC-Cy7-conjugated anti-EpCAM (clone G8.8;#118218, 1:100, Biolegend), PE-conjugated anti-CD51 (rat, clone RMV-7, Biolegend #104106, 1:50), BV421-conjugated anti-CD61 (Armenian hamster, clone 2C9.G2, BD Bioscience #553345, 1:50), biotin-conjugated anti-CD106 (rat, clone 429 (MVCAM.A), BD Bioscience, #553331, 1:50), BV711- conjugated anti-Epcam (rat, clone, G8.8, BD Bioscience, #563134, 1:100, PerCPCy5.5 conjugated anti-CD45 (rat, clone 30-F11, BD Bioscience, #550994, 1:100), and PerCPCy5.5 conjugated anti-CD31 (rat, clone MEC13.3, BD Bioscience, #562861, 1:100), PE Anti- Active Caspase-3 (BD Pharmingen #550821, 1:25) and PE Anti- H2AX (pS139) (BD pharmingen #562377, 1:20), Alexa Fluor 647 anti-BrdU (BD Pharmingen #560209, 1:50). Validated for flow cytometry by manufacturer.
Immunoprecipitation were performed using 6µg of rabbit Igg control Chip grade (Abcam, ab171870) ; rabbit IPO9 (A305-475A , Bethyl Lab). Validated for immunoprecipitation by manufacturer.

Validation

Affinity-purified mouse monoclonal antibodies NB8-AD9 raised against human phospho-CDK4 (Thr172) was characterized in Coulonval et al. (2022) 10.1080/15384101.2021.1984663 and allow the direct detection of endogenous phospho-CDK4 from human and mouse cells by a variety of techniques including immunoblotting, immunoprecipitation.
The other antibodies used are commercially available and were validated by the provider. We used protocols and recommandations of the manufacturer on validated species.

Eukaryotic cell lines

Policy information about cell lines

Cell line source(s)

Primary mouse skin SCC cell lines isolated from Lgr5/Kras/p53 RhoJ WT and RhoJ cKO skin SCC, human MDA-MB-231 cell line (ATCC HTB-26)

Authentication

the cell lines have not been authenticated

Mycoplasma contamination

All cell lines have been tested negative for mycoplasma contamination

Commonly misidentified lines
(See ICLAC register)

No commonly misidentified cell lines were used in this study according to ICLAC register version II.

Animals and other organisms

Policy information about studies involving animals; ARRIVE guidelines recommended for reporting animal research

Laboratory animals

Rosa26-YFP, Rosa-tDTomato, K14CreER , Lgr5CreER, KRasLSL-G12D and p53fl/fl mice have been imported from the NCI mouse repository and the Jackson Laboratories. RhoJ fl/fl mice were a kind gift from A. Uemura (Department of Retinal Vascular Biology, Nagoya City University Graduate School of Medical Sciences, Japan). All mice used in this study were composed of males and females with mixed genetic background. Mouse colonies were maintained in a certified animal facility in accordance with the European guidelines. All the experiments were approved by the Ethical Committee for Animal Welfare (Commission d' Ethique et du Bien Etre Animal, CEBEA, Faculty of Medicine, Université Libre de Bruxelles, reference no. 434N and 663N).
Lgr5CreER/Kras/p53/RYFP, Lgr5CreER/Kras/p53/RHOJ KO/RYFP, Lgr5CreER/Kras/p53/tdTomato and Lgr5CreER/Kras/p53/RHOJ KO/tdTomato were induced with tamoxifen at 28-35 days after birth. Tumor appearance and size were detected by daily observation and palpation. Mice were euthanized when tumor size reached 1cm3 or when mice presented signs of distress or lost >20% of its initial weight. For grafting experiments, NUDE mice were used with age ranging from 4 to 8 weeks.

Wild animals

No wild animals were used in this study

Field-collected samples

No field-collected samples were used in this study

Ethics oversight

Mice colonies were maintained in a certified animal facility in accordance with European guidelines.The experiments were approved by the local ethical committee (CEBEA) under protocols 434N and 663N. The study is compliant with all relevant ethical regulations regarding animal research.

Note that full information on the approval of the study protocol must also be provided in the manuscript.

Flow Cytometry

Plots

Confirm that:

☒ The axis labels state the marker and fluorochrome used (e.g. CD4-FITC).

☒ The axis scales are clearly visible. Include numbers along axes only for bottom left plot of group (a 'group' is an analysis of identical markers).

☒ All plots are contour plots with outliers or pseudocolor plots.

☒ A numerical value for number of cells or percentage (with statistics) is provided.

Methodology

Sample preparation

Tumors were dissected, minced and digested in collagenase I (Sigma) during 2 hr at 37°C on a rocking plate. Collagenase I activity was blocked by the addition of EDTA (5 mM) and then the cells were rinsed in PBS supplemented with 2% FBS. Before the staining, cells were blocked during 20 min at room temperature in PBS supplemented with 30% FBS. Cell suspensions were filtered through a 70 µm cell strainers (BD) then through a 40 µm cell strainer to ensure the elimination of cell debris and clumps of cells.

Instrument

FACSaria III and LSRFortessa (BD Bioscience)

Software

FACSDiva Softwara 8.0.1 (BD Bioscience) and FlowJo v10

Cell population abundance

The proportion of YFP+ tumor cells in Lin- population varied from 20 to 95%. The proportion of tumor cell subpopulations within YFP+ tumor cells varied depending on the tumor type and between individual tumors.

Gating strategy

Living cells were selected by forward scatter, side scatter, doublets discrimination and by Hoescht dye exclusion. Tumor cell subpopulations were selected based on the expression of YFP or Tomato and the exclusion of CD45 and CD31

☒ Tick this box to confirm that a figure exemplifying the gating strategy is provided in the Supplementary Information.
